# Articulation Morphology of Plants and Plant Evo-Devo: An Open Morphology—Empirical, Dynamic, All-Inclusive, and Unifying

**DOI:** 10.3390/plants15050730

**Published:** 2026-02-27

**Authors:** Rolf Sattler

**Affiliations:** Biology Department, McGill University, Montreal, QC H3A 0G4, Canada; sattler.rolf@gmail.com

**Keywords:** plant morphology, plant evo-devo, plant morpho evo-devo, homology, open growth, ramification, anaphytes, process morphology, complementarity, philosophy of plant morphology

## Abstract

In Articulation Morphology, inspired by the theory of anaphytes that was first proposed in 1843, ramification is the key principle in plant morphology in the open growth of plants. It engenders articulation: the formation of articles, called anaphytes. While the theory of anaphytes included tenets that are now considered outdated, Articulation Morphology—proposed here as a modern version of this theory—retains and further develops only those aspects that remain valid and fundamentally important, namely ramification and articulation. In this view, plants are articulated wholes: systems of articles formed through ramification and articulation: the formation of articles. These articles are understood dynamically as process combinations according to process morphology. For practical purposes, they may be described in traditional structural terms such as root, stem, leaf, or leaflet, but without implying a controversial and limited morphological theory such as the classical root–stem–leaf theory of mainstream morphology. Hence, articulation morphology is strictly empirical, solely relying on the observable processes of open growth, ramification and articulation. In contrast to classical mainstream morphology, which often fails to accommodate atypical or deviant structures, articulation morphology is all-inclusive: even the most deviant structures can be understood as deviant patterns of ramification and articulation. Furthermore, articulation morphology is unifying because articles constitute a fundamental morphological unit that applies to all plants from algae to bryophytes and vascular plants, whereas organ-centred classical mainstream morphology lacks such a fundamental unifying unit for all plants. Within this framework, the central concept of articulation morphology is no longer *homology* but *transformation*—the transformation of ramification and articulation. Owing to this fundamental shift and to its empirical, dynamic, all-inclusive, and unifying foundation, articulation morphology may be regarded as a new paradigm for plant morphology—an open morphology. From this perspective, plant evo-devo, especially plant morpho evo-devo, becomes the investigation of the development and evolution of ramification and articulation.

## 1. Introduction

Classical morphology remains predominant in mainstream plant morphology [1,2], although it has been surpassed (e.g., [2,3,4,5]). According to classical morphology, plants such as flowering plants consist of three kinds of organs: root, stem (caulome), and leaf (phyllome) [1,2,6,7]. Thus, any organ we encounter must be interpreted as either a root, a stem (caulome), or a leaf (phyllome). However, some structures deviate so markedly from the common pattern that they cannot be clearly assigned to any one of these three organ categories [5,8,9]. As a result, persistent debates have arisen about the categorical assignment of such controversial structures. These controversies remain unresolved because they amount to pseudo-problems: attempts to categorize structures that do not fit the categories (for examples, see below). Articulation morphology offers a way out of this impasse. Inspired by the theory of anaphytes (anaphytosis), it may be regarded as a modern extension of this theory. It is grounded in the open growth of plants, which entails ramification and articulation: the formation of articles (segments such as internodes and simple leaves). Because it rests on the observable processes of ramification and articulation, articulation morphology is inherently empirical and dynamic. By accommodating all structures, including those that do not fit within the categorical framework of classical mainstream morphology, it is all-inclusive. Through the concept of the article (or segment), which applies to all plants and all levels of organization, it achieves a unification lacking in classical mainstream morphology. In this way, articulation morphology frees us from the constraints of the categorical organ-centred view that has long dominated mainstream morphology.

After outlining the theory of anaphytes, I introduce articulation morphology and highlight its significance and advantages over classical mainstream morphology. I then relate articulation morphology to other morphological approaches and discuss the relevance and irrelevance of homology, as well as the fundamental assumptions underlying all morphological approaches. And finally, I examine how articulation morphology relates to evo-devo.

## 2. The Theory of Anaphytes (Anaphytosis)

In the theory of anaphytes (anaphytosis), developed by C. H. Schultz, who is also known as Schultz-Schultzenstein, pseudo-problems of classical mainstream morphology do not arise [10,11]. According to this theory, plant morphology results from two fundamental processes: ramification (branching) and articulation. Both processes are directly observable. We can observe that during the development of plants, ramification occurs and leads to articulation: the formation of articles, called anaphytes, which arise after each ramification and between successive ramifications. For example, a simple leaf is an article that does not undergo further ramification, whereas an internode is an article between successive ramifications. The continued formation of anaphytes is called anaphytosis; hence, Schultz-Schultzenstein named his theory anaphytosis. I refer to it more simply as the theory of anaphytes. Besides the fundamental processes of ramification and articulation, this theory also comprised theoretical claims that today appear outdated. Anaphytes were regarded as individuals capable of forming a whole plant. I do not endorse this claim and other speculative components of this theory. I retain only its factual basis of ramification and articulation.

The theory of anaphytes has been almost completely forgotten. Only very few authors have referred to it [12,13]. Adrian S. Foster, originally the senior author of the well-known textbook *Comparative Morphology of Vascular Plants* [14], concluded late in life that “a theory, in some way analogous to that of the anaphytes, was most valuable” (quoted in [12] (p. 46). Nevertheless, Kaplan [1], one of his students, like most mainstream morphologists, ignored—or seemed unaware of—the theory of anaphytes and remained committed to classical morphology, although the morphological foundation of the theory of anaphytes provides a more inclusive and comprehensive framework.

## 3. Articulation Morphology

Organisms can be partitioned into different kinds of parts [15]. Thus, plants can be partitioned into organs or articles (anaphytes). Organs are established, if not through questionable non-observable boundaries, then at least through a morphological theory or a model such as the classical root–stem–leaf model and its associated homology. In contrast, an article arises from the observable initiation of a new growth centre (primordium). Hence, it is empirically defined. The organ-based approach of mainstream classical morphology has been solidified by the prevalent idea that plants consist of three fundamental organs (“Grundorgane” in German), as promoted by Troll, the influential German morphologist [16,17] and later by Kaplan, the influential American morphologist, who, like most mainstream morphologists, adopted Troll’s categorical framework [1,2]. In contrast to the organ-based approach of mainstream morphology, according to articulation morphology—a modern version of the theory of anaphytes that I am proposing here—plants are articulated (or segmented) wholes: systems of articles generated by open growth, which leads to ramification and articulation: the formation of an article from each ramification and between successive ramifications. In this view, open growth, ramification, and the formation of articles constitute the most fundamental processes in plant morphology and plant evo-devo. Instead of ‘articles,’ one could refer to segments, structural units or simply structures defined by ramification and subsequently differentiated in many ways. These include thallus segments, telomes, roots, segregation products of the shoot apical meristem (SAM) arising from lateral and axillary meristems, fractionation products of the reproductive apical meristem (RAM), and rare structures such as the fronds of the Lemnaceae and haustoria (see below).

Morphology based on ramification and articulation could be called ramification morphology or articulation morphology. I prefer the latter because it emphasizes more strongly the difference to mainstream morphology, which also addresses ramification but often in a more restrictive way concerning the formation of branches such as axillary branches. In contrast, the process of ramification in articulation morphology refers to the formation of new primordia regardless of which articles they produce. Hence, in articulation morphology, ramification is used in a much broader and more fundamental sense than in mainstream morphology. Thus, structures such as compound leaves, stamens and carpels are also understood as ramified.

Articulation morphology can also be called an *open morphology* because it is based on open growth as the source of ramification and articulation and recognizes plasticity as a correlate of open growth. Furthermore, *open morphology* can also be understood as being open toward other complementary approaches to morphology.

Openness implies continuity, but besides implying a morphological theory and homology, mainstream morphology often invokes demarcations between organs that are questionable because there are no clear-cut boundaries between organs understood as morphological body parts [18]. By drawing boundaries differently, five different models of plant construction have been distinguished [5] (pp. 487–489). These models complement one another, yet they are all based on boundaries that do not exist in nature. The shoot—and indeed the whole plant—is a continuum. Within this continuum, Howard [19] highlighted the stem-node-leaf continuum. This continuum is acknowledged in the model that subdivides the plant into phytomers, which are also called modules [5,20] However, phytomers—consisting of a node, the internode below, the leaf, the axillary bud and even roots if present—create a discontinuum in the stem from one internode to another. This discontinuum is avoided in the traditional root–stem–leaf model by acknowledging the continuity of the stem, but at the cost of drawing a boundary between the stem and the leaf. Other models of the shoot avoid one discontinuum only to create another. In contrast, articulation morphology is not based on organs with disputed boundaries; it is based on articles—units that emerge through the observable process of ramification. They are not defined by boundaries; we simply observe that ramification occurs through the formation of a new growth centre (primordium), which at its base is continuous with the article on which it is formed. As this new primordium develops into a new article, it acquires distinct properties and through these properties—not through a non-existent boundary—it becomes distinguishable from the article on which it arises. Hence, the distinction of articles does *not* rely on boundaries: we can indeed distinguish articles without drawing boundaries. If the newly formed article does not ramify, it remains a single article. If it does ramify, the segment between successive ramifications constitutes an article. Because articulation morphology does not rely on boundaries that do not exist in nature, it surpasses the complementarity of the five shoot models that are based on different, complementary boundaries.

Even between organs, no boundary can be observed. Yet in mainstream morphology, a boundary is often assumed, but some mainstream morphologists acknowledge its non-existence. Kaplan wrote: “The vagaries of defining the boundaries between stem and leaf components of the shoot underscore the fundamental developmental unity of these two elements and the artificiality of attempting to draw a rigid boundary between them” [1] (p. 5). Nonetheless, Kaplan (ibid., p. 192) refers to leaf bases surrounding the stem—as in the leaf skin model—and he insists that the shoot “consists of two major components: leaves and stem” (ibid., p. 4) and that “a flower is a reproductive shoot bearing microsporophylls (stamens) … and megasporophylls (carpels) as its appendages or leaf homologues” (ibid., p. 1069). Thus, even if the attempt to draw boundaries is given up, a crucial difference between organ-based mainstream morphology and articulation morphology remains: in mainstream morphology, organs are defined in terms of a *morphological* theory and homology, whereas articulation morphology does not depend on a morphological theory and homology; it is solely based on the observable processes of ramification and articulation, independent of any morphological theory and homology. Therefore, it avoids the shortcomings and pseudo-problems of the classical theory, which cannot account for structures that do not fit into its categorical framework. Claβen-Bockhoff [5] avoids these pseudo-problems by acknowledging that extreme forms cannot be accommodated by mainstream morphology. However, she retains organs as the fundamental morphological units, and organs are delimited by the classical theory and homology. Thus, a compound leaf is an organ homologous with a simple leaf. This tenet has led to seemingly unresolvable controversies [21,22,23]—controversies that are surpassed by articulation morphology because it is not based on organs but on the observable processes of ramification and articulation. We thus can observe that a compound leaf is a system of articles, whereas a simple leaf represents only a single article. The question whether a compound leaf is homologous with a simple leaf does not arise or is secondary.

In articulation morphology—which also could be called segmentation morphology—the central and most basic concept is no longer morphological homology but transformation: the transformation of ramification and articulation. This changes the most basic questions we ask. Instead of asking questions about morphological homology, we ask how ramification and articulation have changed during development and evolution. For this reason, articulation morphology may be considered a new paradigm of plant morphology. It fundamentally changes our way of thinking about morphology and, consequently, morphological investigation.

In evo-devo, evolution is seen as the transformation of ontogenetic development. Morpho evo-devo focuses on morphological transformation. In terms of articulation morphology, this means that during development (ontogeny) and evolution (phylogeny), articles are added, eliminated, or replaced by others that are more or less different. To investigate this transformation—that is, the evolution of development (ontogeny)—we do not need homology, because articulation morphology investigates transformation directly without the interference of morphological homology, especially categorical (classificatory) homology in terms of morphological categories. Thus, the problem or pseudo-problem of the categorical assignment of controversial structures does not arise in articulation morphology. However, if one wishes to compare articles, it may be done using fuzzy set theory, where differences range from 0% toward 100%. 0% means no difference, hence sameness or identity. If one does not or cannot quantify the difference, one can refer to “more or less different,” or “more or less similar”. One could interpret the difference or similarity as the homology of articles, ranging from total to partial or combinatorial homology [3,24,25]. However, as emphasized above, the primary aim of articulation morphology is not morphological homology but transformation. Fossils, biogeography, ecology and developmental genetics may provide data that help to determine the direction of transformation during evolution. Other types of homology—beyond the morphological—may also play a role in the elucidation of evolution [26,27].

How do we describe articles? I propose describing articles as process combinations according to process morphology [3], which includes processes such as growth duration and growth distribution resulting in radial, bilateral, or dorsiventral growth (see [28]). Articles may differ in growth duration and may arise as radial, bilateral, or dorsiventral primordia. In the latter case, they may be oriented in the same plane as the article on which they are formed or in a transversal plane, that is, in a plane perpendicular to the plane of the parent article. For example, teeth in serrated leaves arise in the plane of the leaf, whereas leaflets may be formed in a plane perpendicular to that of the leaf [21,22].

For convenience, familiar terms may be used for the process combinations, and new terms may be coined when necessary. For example, the process combinations that constitute what we commonly call a simple leaf may be referred to as a simple leaf as long as we keep in mind that a leaf is not a static structure but a process combination. Other terms of mainstream morphology, such as root and internode, may also be used, and thus, a limited continuity between mainstream morphology and articulation morphology is possible.

However, the difference between mainstream morphology and articulation morphology is more fundamental than may initially seem. Whereas some articles correspond to organs of mainstream morphology, others do not. For example, a simple leaf, an internode, or a root is equivalent to an article because they result from a single ramification. However, a compound leaf, which is considered one organ according to mainstream morphology, is a system of articles. In flowers, the difference between mainstream morphology and articulation morphology is even more striking: stamens and carpels are systems of articles, not leaf homologues.

Because ramification is fundamental to articulation morphology, we have to distinguish different modes of ramification: dichotomous, lateral, and axillary ramification. Dichotomous ramification is common in thalloid liverworts such as *Marchantia* and the earliest telomic fossils such as *Rhynia*. Whereas the articles in thalloid liverworts are dorsiventral, those in the earliest telomic fossils are mostly of radial symmetry, but a continuum from radial to dorsiventral symmetry has been documented [28]. In the telomic fossils, it is most obvious that they consist of articles. An article arising from a single ramification that does not ramify further is called a telome, whereas an article between two successive ramifications is called a mesome. Both telomes and mesomes are telomes in the broad sense. Thus, a telomic fossil is a telome truss that consists entirely of telomes in the broad sense.

According to the telome theory [29,30,31], the diversity of vascular plants has evolved through elementary processes that produced different patterns of ramification and articulation. Patterns are sequences of ramification and articulation. The elementary process of overtopping leads from dichotomous to lateral ramification, whereas the elementary process of planation describes the change from three-dimensional to two-dimensional ramification. The elementary process of fusion has been invoked in leaf formation, which has, however, led to criticism [32,33]. But Zimmermann himself [30] (p.105) [34] pointed out already that “fusion” should not be understood as an actual fusion but rather as a basipetal shift in growth.

To the extent that the telome theory explains the diversity of vascular plants, it corresponds to articulation morphology because telomes are articles, not organs. However, the telome theory is more limited than articulation morphology because it does not address plant structures of bryophytes and algae, and it is difficult or impossible to apply to more highly evolved vascular plants, such as seed plants, in which individual telomes are usually no longer recognizable. Moreover, intercalary meristems leading to zonal growth play an important role, especially in flowers. For example, inferior ovaries may be formed through intercalary growth. Interprimordial growth leads to a continuity between primordia (often described as “fusion”), as, for example, in the formation of a sympetalous corolla.

Whereas dichotomous ramification is rare in vascular plants, lateral ramification is widespread and axillary ramification is characteristic of seed plants. Acknowledging this, Sachs [35] proposed a schematic ground plan of seed plants, which was adopted by classical morphologists such as Troll [7] and Kaplan [1]. It comprises a root system and a shoot system with a stem, leaves, and axillary buds. Hence, it represents the trinity of the classical organ categories: root, stem (caulome), and leaf (phyllome). It applies only to a limited extent.

There is evidence for a continuum from roots to shoots, stems, leaves, leaflets, stipules, enations, and hairs [21] (pp.82, 139–141), [36] (Figure 39). Articles and systems of articles span this whole continuum. Since hairs are often formed much later than the more prominent structures, we may distinguish two phases of ramification—an initial phase of the more prominent structures, traditionally often referred to as organogenesis, and a later phase of hair formation—but the two phases may overlap through enations (emergences). Stem scales in the African genus *Inversodicraea* of the Podostemaceae [37], also sometimes referred to as leaves, are not vascularized and thus can be considered enations (emergences). They are inserted irregularly around the stem, whereas the vascularized leaves are arranged in a distichous order. Recognizing the continuum between different morphological levels of organization—from hair (trichome) to organ to the organ system of the shoot—frees us from the organ-centred approach of mainstream morphology.

For a more complete description of ramifications, the spatial arrangement of articles has to be taken into account. If we refer to phyllotaxy in articulation morphology, this notion must be understood in a wider sense: not only as the positioning of leaves on the shoot apical meristem (SAM) but as the arrangement of all articles. These articles may arise sequentially (as in spiral phyllotaxy) or simultaneously (as in whorled and decussate phyllotaxy). Deviations from these patterns occur, especially in flowers [5].

Any particular plant, or group of plants, may be described by its sequence of ramifications and the resulting articulations. Thus, articulation morphology, based on open growth, encompasses the whole life cycle of plants. In seed plants, the embryo is typically bipolar, differentiating into two opposite poles: the root pole and the shoot pole (for exceptions see [5] (pp. 599–600)). The root pole develops into the main root, from which side roots arise through ramification and articulation. One, two, or more cotyledons (articles) arise from the apical region of the embryo lateral to the shoot pole. The hypocotyl (representing one article) is formed between the cotyledon(s) and the first side root. It unifies the two poles, creating oneness in duality. The shoot pole develops into the shoot apical meristem (SAM). As it ramifies through lateral and axillary meristems, it also forms internodes, each representing one article. Lateral meristems usually develop into simple leaves (each consisting of one article) or compound leaves (each comprising a system of articles). Occasionally, uncommon structures (representing one or more articles) occur (see below). Axillary meristems normally produce a side branch, generated by a new SAM, but occasionally also other structures, such as phylloclades (consisting of many or only one article), occur in the axillary position and rarely even the SAM may become transformed into a phylloclade (see below). Usually, much later, hairs—minute articles—may be formed on various structures. Furthermore, common (typical) and uncommon (atypical) structures (consisting of one or more articles) may arise in deviant positions. For example, typical roots may be formed on the hypocotyl, and highly deviant structures such as haustoria may develop on roots, stems, and leaves [5] (pp. 641–647). As in Daoism, where Yin comprises Yang and vice versa, the root retains the potential to develop shoots, and the shoot may form roots.

In angiosperms, the SAM eventually becomes transformed into an inflorescence meristem, a floral unit-meristem, or directly into a floral meristem [5]. Usually, the first ramifications of the floral meristem produce the members of the perianth, each corresponding to one article, followed by the sporangiophores, which consist of a system of articles [5].

Pteridophytes and bryophytes also exhibit polar organization. In addition to a shoot, most Pteridophytes have roots, but they are not derived from an embryonic root pole opposite the shoot pole, as in seed plants. Even plants lacking roots are polarized. Thalloid liverworts such as *Pellia epiphylla* exhibit vertical polarity: the lower side of their dorsiventral thallus faces the earth and its upper side the sky. In addition, the thallus is polarized in the horizontal plane: it grows at the anterior end while decaying at the posterior, thus displaying a life and death pole. This dynamic grants it the potential for immortality. Contrary to most plants, these plants are not sedentary: as one end continues to grow, it slowly and imperceptibly moves across the surface of the ground.

The earliest fossil land plants probably had a dorsiventral thallus resembling that of thalloid liverworts, growing horizontally close to the ground. Subsequently, this prostrate growth became increasingly vertical (erect), as seen in extant liverworts such as *Hymenophyton flabellatum* [38] (p. 62) and the telomic plants. This shift toward verticality was crucial for the evolution of the morphological diversity of vascular plants, culminating in the towering trees of seed plants, spanning ground and sky, earth and heaven, which the Chinese Daoists understand as an expression of Yin and Yang. According to Hagemann [39,40] and Schilperoord [38], fossil liverworts gave rise to vascular plants, whereas the predominant view holds that vascular plants evolved from fossil telomic plants [34]. Sattler [28] proposed a synthesis of these opposing views. As articles develop, they acquire many traits. One important and widespread trait is surface expansion, which can lead to flattened structures such as leaves and leaflets.

Research in plant architecture complements articulation morphology, especially with regard to higher-level units such as branches and the plant as a whole. The different architectural models of trees [41,42] can be understood as strange attractors that are inherently fuzzy, thus requiring fuzzy logic for their proper analysis [43].

Because of its wide applicability and practical terminology, classical mainstream morphology also remains useful. However, it is limited in scope, as it cannot adequately account for structures that do not fit its categories. These structures have been referred to as “misfits” [4,44,45,46]. As Bell [44] emphasized, misfits are “misfits to a botanical discipline [such as classical morphology], not misfits for a successful existence”.

One example of such misfits is phylloclades, such as the sterile phylloclade of *Semele androgyna* of the Asparagaceae [47]. It is a dorsiventral axillary structure whose homology has been debated for centuries. However, for articulation morphology, it is unproblematic: it simply represents a dorsiventral article. In this framework, we do not ask whether this dorsiventral article is homologous with a leaf or a branch. Instead, we ask how development has changed during evolution (evo-devo). If the ancestors of *Semele* bore axillary branches, then during evolution these branches (a system of articles) have been replaced by a single dorsiventral article—except in the fertile phylloclades, where the article bears inflorescences (systems of articles). Thus, in articulation morphology, the question of homology does not arise. Endless debates about the homology of these structures are not helpful and appear futile because they are based on a pseudo-question: the assumption that a structure that does not fit the categories must nonetheless be forced into them. In *Ruscus aculeatus,* in addition to axillary phylloclades, terminal phylloclades occur: the shoot apical meristem (SAM) is directly transformed into a dorsiventral structure that resembles a leaf [47].

Another example of a misfit is found in *Chisocheton tenuis* (Meliaceae)*,* where lateral structures in leaf positions exhibit indeterminate growth and bear inflorescences and vegetative shoots on their adaxial side [48]. Again, the homology of these structures has been debated for a long time without any final resolution (ibid.). Transcending homologization, articulation morphology understands them simply as an elaborated system of articles resembling pinnate leaves but with indeterminate growth. Thus, during evolution, pinnate leaves acquired indeterminate growth that is typical of shoots.

Even typical pinnate leaves remain controversial [21,22,23,49]. They too cannot be clearly fitted into the leaf category. But from the perspective of articulation morphology, they simply represent an article that produces a secondary set of articles in a distichous arrangement. This articulate view is significant because it supersedes the persistent debate over whether a compound leaf constitutes a single organ homologous with a simple leaf or a shoot or partial shoot. Nonetheless, if desired, questions of homology may be considered. Some molecular geneticists have assumed that morphological homology may be inferred from gene expression (e.g., [50]). However, a structure such as a compound leaf is much more than genes. It cannot be reduced to genes alone [4], although gene expression is relevant and may even be compatible with morphological homology—for example, by suggesting a partial homology between at least some compound leaves and shoots. In this way, gene expression may also be relevant to the issue of morphological misfits.

An extreme example of a morphological misfit is *Wolffia arhiza*, the smallest vascular plant on Earth. It forms flattened, rootless structures, often referred to as fronds [51]. Homologization seems impossible and unnecessary. As Wake [52] pointed out, debates over homology—in this case, whether the frond is homologous to a stem or leaf—are irrelevant to understanding developmental transformation during evolution and consequently distract from this central issue. Because the developmental transformation of *Wolffia* fronds deviates drastically from the typical pattern of flowering plants, Claβen-Bockhoff [5] (pp. 354–355) included the fronds among extreme forms that do not fit the categories of classical morphology, and Minelli [45] accordingly referred to them as misfits. Yet for articulation morphology, the fronds of *Wolffia* are not misfits but merely uncommon patterns of articulation. There are no misfits in articulation morphology—everything fits.

In addition to being all-inclusive, articulation morphology is also unifying because the concept of the article applies to all plants—from algae to bryophytes and vascular plants—and across all levels of organization from organ systems to organs, enations, and hairs (trichomes). It even applies to the continuum that links these levels of organization. Thus, articulation morphology transcends organ-centred mainstream morphology, which imposes an artificial division between organs and other structures such as stipules, enations, and hairs.

The principal advantages of articulation morphology can be summarized as follows:

1. Articulation morphology, with its central concept of the article, is all-inclusive and thus accounts for the whole diversity of plant form, whereas classical mainstream morphology encounters difficulties and pseudo-problems when dealing with misfits.
2. Articles are directly observable, being empirically defined as the products of ramification, whereas organs depend on a morphological theory and assumptions of homology, such as those underlying the classical root–stem–leaf model.
3. Articles are continuous with one another and thus do not rely on non-existent boundaries for their demarcation. Organs are also continuous, but they are often delimited by boundaries that do not exist in nature. For example, the definition of a “leaf” differs depending on whether one adopts the boundaries implied by the root–stem–leaf model, the leaf skin model, or the metameric model.
4. Articles occur in both the thalli and the cormus, whereas organs are confined to the cormus. Thus, the notion of the article provides a unifying unit for all plants, which is lacking in mainstream morphology.
5. Articles span all levels of morphological organization, from the organ system of the shoot to organs and parts of organs, including enations and hairs (trichomes), whereas mainstream morphology is more limited because it remains organ-centred.
6. Articles are not confined to mutually exclusive categories, whereas organs are traditionally classified into mutually exclusive categories such as root, stem or leaf.

Despite these differences between articulation morphology and categorical organ-centred mainstream morphology, the gulf between the two could be partially bridged through the following adjustments in categorical organ-centred morphology:

- Recognizing more widely that, like articles, organs cannot be demarcated by boundaries, since there is a continuum within a plant between organs such as the stem and leaf. This would render unnecessary the distinction of different shoot models based on different boundary placements (as explained above).
- Acknowledging that some organs, such as compound leaves, constitute a system of structures in which one structure gives rise to other structures, commonly called leaflets. Although “leaflet” literally means “small leaf,” mainstream morphology defines a leaflet as a segment of a leaf. Apart from considerations of homology, recognizing the equivalence of “segment” and “article” could provide a conceptual bridge between the mainstream view and articulation morphology.
- Accepting that mutually exclusive organ categories are too rigid to encompass the full diversity of plant forms. If homology is still pursued, allowing, alongside categorical total homology, also partial homology [3], factorial or combinatorial homology [24,25,53]. Thus, using fuzzy logic in addition to Aristotelian identity and either/or logic.

As an alternative to mainstream morphology, one may redefine the leaf in an inclusive sense that transcends classical categories. In his botanical notes from his Italian journey, Goethe suggested this broader perspective with the hypothesis “Alles ist Blatt” (All is leaf). Thus, a leaf that absorbs water we call a root, and a leaf of radial symmetry we call a stem (for the exact German quote, see Schad [54] (p. 211). Schad (ibid.) expressed this broader view by defining the leaf as “jedes potenzreiche Grundgewebe” (any ground tissue rich in potential). In this sense, all plant structures, including those of algae, can be understood as leaves (Schad, personal communication). As Goethe pointed out, the simplicity of just one fundamental unit (instead of two or three basic categories) makes possible the greatest diversity [55] (p. 80). Arber’s partial-shoot theory is also based on only one unit, the shoot, total or partial. Similarly, articulation morphology posits only one unit, the article. However, in contrast to Goethe’s hypothesis and Arber’s theory, the concept of the article is not founded on a hypothesis or theory but on the observable process of ramification that leads to articulation. In this sense, the diversity of plant forms arises through the differentiation of one basic unit, the article, leaving no exceptions or misfits. Consequently, it is no longer necessary to ask whether all structures are homologous with one or another mutually exclusive category, such as root, shoot, stem and leaf. Aristotelian either/or logic—where it leads to pseudo-questions—is thereby transcended.

Articulation morphology—as an open morphology—reveals unity in diversity at both the individual and transindividual levels. At the individual level, this unity resides in the continuity of diverse articles, which together constitute an articulated wholeness or oneness. At the transindividual level, the concept of the article discloses the unity underlying the diversity of all plants. The alternation of generations further unifies the diversity of plants through the continuity of life cycles during evolution. Finally, the continuity of diverse plants with soil and air—in Daoism referred to as earth and heaven, or Yin and Yang—expresses the unity of the ecosystem, which ultimately points to the unity of the differentiated universe as a whole.

## 4. Articulation Morphology in Relation to Ten Approaches to Plant Morphology

To better understand articulation morphology, it must be situated within the broader landscape of morphological approaches. Accordingly, I will briefly outline differences and commonalities between articulation morphology and other approaches to plant morphology, discuss the relevance or irrelevance of homology, and indicate how articulation morphology even surpasses continuum and process morphology as envisaged previously, the two approaches most closely related to it. Finally, I will examine the common basis of all morphological approaches.

1. Classical Morphology

Classical morphology asserts that all structures, at least in seed plants, can be understood in terms of the root–stem–leaf model or the metameric (modular) model, according to which seed plants consist only of roots and leaves that comprise the stem segments. This means that any organ we encounter must be homologous with either a root, a stem, or a leaf, or only a root or a leaf (e.g., [1,2]). However, the categorization of atypical structures remains controversial and may lead to pseudo-problems, as pointed out above. In articulation morphology, such pseudo-problems do not arise.

Articulation morphology is compatible with classical morphology to the extent that articles coincide with organs such as roots or simple leaves. Otherwise, articles are more inclusive than organs, as noted above. Nonetheless, classical morphology can be considered complementary to articulation morphology because, although more limited, it seems appropriate and useful for the majority of structures and provides convenient terminology.

Plant architecture [41,42] may be seen as an extension of classical morphology. Because it focuses on higher-level units such as branches and the whole plant, it may encounter the problems or pseudo-problems of classical morphology only rarely.

2. Evolutionary Developmental Morphology (Evo-Devo Morphology)

Evolutionary developmental morphology (according to [5]) explains the majority of vegetative structures in terms of the basic organs of classical morphology, but—like Rutishauser [4]—recognizes the existence of extreme forms (misfits according to Rutishauser) that cannot be fitted into the classical categories. As pointed out above in connection with the fronds of *Wolffia*, for articulation morphology, such extreme forms present simply unusual patterns of articulation.

In evolutionary developmental morphology, flowers are considered *de novo* structures to which the categories of classical morphology do not apply. Again, this poses no problem for articulation morphology.

3. Arber’s Partial-Shoot Theory

Arber’s Partial-Shoot Theory recognizes only one morphological category: the shoot—total or partial [21]. According to this theory, structures such as compound leaves, simple leaves, and roots are partial shoots (ibid., 132–135, 140–142, 159). Because partiality is a matter of degree, Rutishauser and Isler [36] introduced the term *Fuzzy Arberian Morphology (FAM)*, implying fuzzy logic in contrast to the either/or logic of classical morphology (ClaM). Fuzzy logic is compatible with articulation morphology. However, because Arber’s theory applies only to plants with shoots and therefore excludes hornworts, thalloid liverworts, and algae, it is more limited than articulation morphology.

4. The Telome Theory

Since telomes coincide with articles, the telome theory [29,30,31] is compatible with articulation morphology. However, because the telome theory deals only with vascular plants, it is more limited than articulation morphology, which also applies to bryophytes and algae. Furthermore, since the telome theory cannot be well applied to most seed plants, where telomes are no longer easily recognizable, it has further restrictions that do not occur in articulation morphology.

5. Continuum Morphology

Continuum Morphology [3] has two aspects: 1. A continuum within any individual plant, since there are no clear-cut boundaries between parts of a plant, such as roots, stems, and leaves, and 2. A continuum between the structural categories of classical morphology. This continuum spans not only the organ categories but also the different morphological levels of organization from hair to organ to the organ system of the shoot, thus moving beyond the organ-centred view of mainstream morphology. Extreme forms (misfits) are absorbed into this continuum and therefore cease to be exceptions or misfits. Articulation morphology includes continuum morphology and also goes beyond it. Whereas continuum morphology, as envisaged previously [3], relies on the classical categories as a reference system and demonstrates a continuum between them, articulation morphology operates independently of these classical categories. It can, however, retrospectively reveal that common patterns of ramification and articulation correspond to classical categories. It may also be possible to devise a continuum morphology that does not rely on the classical categories as reference points.

6. Process Morphology

Among all morphological approaches, process and continuum morphology are most closely related to articulation morphology. Process Morphology [3] conceives structures as combinations of morphogenetic processes. Τhese process combinations form a continuum—as the structures in continuum morphology—and for practical purposes, they may be referred to as structures.

Process morphology can be understood in at least two ways:

1. As in continuum morphology, the categories of classical morphology serve as reference points but are understood as process combinations, linked through a dynamic continuum [3].
2. Alternatively, process morphology and process combinations can be understood independently of the reference system of classical morphology. In this broader sense, articulation morphology is a uniquely dynamic approach based on the fundamental process of open growth, which entails the equally fundamental processes of ramification and articulation. It investigates how these processes manifest during ontogeny and how ontogeny has changed during evolution, that is, articulation morphology operates within the evo-devo framework.

Process morphology, as previously envisaged [3], did not address ontogeny in its entirety but only compared typical and atypical process combinations, demonstrating that they are linked through a continuum. Because articulation morphology incorporates ontogeny from the embryo to the mature plant, it constitutes a more comprehensive process morphology.

7. Pictorial Morphology

In pictorial morphology, verbal descriptions are replaced by pictures [56]. Pictures (photos or drawings) provide more information than verbal descriptions. Even a description in terms of process combinations cannot convey the detail presented in a picture. Hence, pictorial morphology complements other morphological approaches, including process morphology.

8. Algorithmic Morphology

Algorithmic Morphology involves modelling, simulation, and visualization of plant development using computational methods, including computer graphics, formal language theory, and programming language design [57,58,59]. It may contribute to the causal analysis of plant development, as, for example, in phyllotaxis research [60,61]. Furthermore, it has considerable aesthetic appeal [62].

Because articulation morphology does not involve algorithms, it differs from algorithmic morphology. However, to the extent that algorithmic morphology generates patterns of ramification, it is compatible with articulation morphology. It remains to be seen how much of the morphological diversity of plants can be explained through algorithms.

9. Causal Morphology

Causal Morphology investigates the causation of plant development on the background of the descriptive and comparative frameworks of the preceding approaches. Nowadays, the predominant causal analysis is through developmental genetics. Numerous genes affecting plant form have been identified, and gene expression may be influenced by epigenetic factors. Even the experimenter may influence gene expression, a phenomenon known as the experimenter effect [63]. Because articulation morphology, as a *morphological* approach, does not encompass molecular biology, causal morphology complements it. However, an extended articulation morphology could potentially be integrated with causal morphology (see below, in the section on Evo-Devo).

10. Functional Morphology

Functional morphology examines the functions of morphological traits. According to Bai [64], the two major functions of plants are the improvement of energy acquisition (photosynthesis) and adaptations to environmental stress. Because this approach relates morphology to ecology and evolutionary theory, it complements articulation morphology; however, the function of articles and systems of articles could also be integrated into an extended articulation morphology. In this context, it has to be recognized that morphology is not simply related to ecology but needs to be adapted continuously to the particular environment in which it occurs. Plasticity plays an important role in this regard.

At least some of the nine approaches overlap, and the list is not exhaustive. The modular approach based on the metameric model could be considered a separate approach [5] (pp. 352, 489); however, it can also be regarded as a version of classical morphology because it was already included in Goethe’s classical Metamorphosis of Plants (1790) [13]. The fertile leaf model [5] (p. 489) is related to the root–stem–leaf model and the metameric model because it interprets the leaf and its axillary meristem as a single unit. The leaf-skin model [5] (p. 489), according to which the leaf bases surround the stem, is part of the classical approach. Plant architecture might be considered a separate approach or an extension of the classical approach. All ten approaches complement one another to various degrees, with articulation morphology as the most inclusive *morphological* approach. Although classical and continuum morphology have been conceived as subclasses of process morphology [65,66], they can also be considered complementary. Articulation morphology is not only complementary to other approaches, but eventually, it might also be integrated with at least some of them. Thus, one might investigate the causation, function and adaptation of articles and systems of articles. For an even more comprehensive understanding of the ten approaches and their relationship to articulation morphology, we must also examine homology.

## 5. Relevance and Irrelevance of Homology

It is important and relevant to discuss homology because it is widely regarded as the most basic and central concept of morphology, the “soul’ of morphology, yet not all approaches imply homology, and there is no consensus on how it should be defined [67]. Since Owen’s [68] classic definition as the sameness of organs, many different definitions have proliferated [27,67]. It is difficult to find a common denominator among these definitions, but one might venture to suggest that they all revolve around ideas of correspondence and similarity or sameness, with or without reference to common ancestry. Throughout this essay, “homology” refers specifically to *morphological* homology, even when not explicitly stated.

How do the ten approaches relate to homology, if at all? In classical morphology and evolutionary developmental morphology (according to [5]), homology is defined as sameness or essential similarity. Thus, if two organs belong to the same morphological category, such as stem or leaf, they are homologous. This kind of homology is based on either/or logic: an organ either belongs to a category or does not. Problems—or rather pseudo-problems—arise when an organ does not fit any of the categories, for example, if it is intermediate between two categories. In such cases, protracted debates have ensued over which of the two categories the organ should be forced into. To end such futile debates, Claβen-Bockhoff [5] acknowledges that there are extreme forms, such as *Wolffia*, that do not fit the classical categories and therefore cannot and should not be homologized with them. Furthermore, she concluded that flowers are *de novo* structures not comparable to the vegetative shoot and that, therefore, the sporangiophores of flowers are not homologous to leaves (phyllomes). This highlights the limitations of the classical homology concept. If, however, we accept the concept of partial, factorial or combinatorial homology [3,25], such misfits can be accommodated as structures that are partially homologous to more than one category. For example, the phylloclade of *Ruscus aculeatus* can be seen as partially homologous to an axillary shoot and a leaf [47]. This combinatorial view of homology is implied in continuum and process morphology [3], and it has also been recognized in causal morphology. Hirayama et al. [69] demonstrated that during the development of the phylloclade of *Ruscus aculeatus*, genes are expressed that are normally expressed in both the shoot apex and leaves. Therefore, these authors concluded that “the phylloclade is not homologous to either the shoot or the leaf, but that it has a double identity” (ibid.). This represents a shift from either/or logic to both/and logic [13], thus providing a more inclusive understanding of homology.

Arber’s Partial-Shoot Theory does not explicitly rely on homology [21]. Because, according to her theory, all structures are whole or partial shoots, the question of whether they are homologous to one or another category does not arise. There is only one fundamental category: the shoot. As such, her theory constitutes a unifying framework like Goethe’s and Schad’s leaf theories (see above).

Similarly, functional morphology and algorithmic approach do not rely on homology. The primary aim of the latter is to generate the widest possible range of morphological patterns. However, once these patterns have been generated, they could be compared in terms of total and partial homology.

In articulation morphology, homology is likewise not foundational. Understanding evolution as changes in ramification and articulation does not require homology; for example, it is not necessary to ask whether compound leaves are homologous with simple leaves or partial shoots. The primary concern is tracing transformations of ramification and articulation. Nevertheless, homology may be considered after the transformations have been analyzed.

To sum up, although homology is still widely regarded as the most basic and central concept of morphology, among the morphological approaches discussed above, Arber’s partial-shoot theory, algorithmic morphology, functional morphology and articulation morphology operate without recourse to homology, whereas evolutionary developmental morphology [5] and causal morphology invoke homology only to a limited extent. Because evo-devo investigates the transformation of development, it does not in itself imply homology, although, to some extent, it has been added as a tool for the investigation of this transformation.

## 6. Fundamental Assumptions of All Morphological Approaches

Despite their differences, morphological approaches share fundamental assumptions. These assumptions are often taken for granted, and many morphologists may not even be aware of them, but they are relevant to the way we understand facts. For naïve realists, facts exist independently of us, whereas for critical observers, facts constitute a consensus that is due to common assumptions. These assumptions are not easily elucidated, but several of their key dimensions can be outlined:

1. Physicality of form—Morphology deals with the physical aspect of plant form. This aspect of form is perceived visually and interpreted intellectually. Therefore, Arber [21,70] understood morphology as a synthesis of the eye and the mind. Materialists maintain that only the physical realm exists, and the mind is seen as an epiphenomenon of the brain—hence, fundamentally also physical. Yet much evidence points to a reality beyond the material, referred to by different names such as spirit (e.g., [71]) or consciousness (e.g., [72,73]). Although morphology is restricted to physicality, it is important to realize that physical form—the very subject of morphology—emerges from a more encompassing reality. Contemplating forms in nature—such as a flower—may lead us to an awareness of this deeper reality.
2. Space and time—The physical aspect of plant form is described and analyzed in terms of the categories of space and time. These categories do not exist independently of us but are our common way of perceiving reality. Mystics and some poets have questioned their ultimate reality. In *Siddhartha*, Hermann Hesse [74] wrote: “Time is not real … And if time is not real, then the dividing line that seems to lie between this world and eternity … is also an illusion”. Similarly, William Blake concluded:
  To see the world in a grain of sand,
  And a heaven in a wild flower,
  Hold infinity in the palm of your hand,
  And eternity in an hour.

Plant morphologists may therefore realize that the common experience of plant form in terms of space and time emerges from a deeper reality of infinity and eternity.

3. Language and abstraction—Morphologists rely on language to describe and analyze plant form. However, words and concepts cannot fully capture reality as it is; they are an abstraction from reality. Abstracting means selecting some features while omitting others. Therefore, Korzybski [75,76] concluded that whatever you *say* about reality is not reality itself. Language functions like a map: useful for navigation, but ultimately not the territory it represents: “the map is not the territory” (Korzybski, ibid.).

How does all this relate to articulation morphology? I emphasized that, in contrast to organs that are based on a morphological theory, articles are factual. However, this does not mean that they represent ultimate reality. An awareness of this limitation can lead to a deeper understanding of plants, from which morphology itself emerges. In this light, contemplating a leaf or a flower may open a doorway to a more profound reality.

Articles of articulation morphology may be seen as an expression of the profound wisdom of the *Heart Sutra*, which, in Tanahashi’s translation [77], states:

Form is boundlessness;
Boundlessness is form.

Since articles are continuous within the whole plant, their form is boundless, yet this boundlessness manifests as the form of articles. Thus, there is no contradiction between boundlessness and articulation, which is indicated by saying that the plant is an articulated whole, a unity in multiplicity, in which form and boundlessness coincide.

Beyond its morphology, a plant extends into its environment. Thus, it is continuous with the soil and the air, the earth and the sky. Ultimately, it is interconnected with everything and thus is integrated into the universe, the most inclusive whole.

## 7. Plant Evo-Devo and Articulation Morphology

According to evo-devo, the evolution of plants is the evolution of plant development [4,26]. Morpho evo-devo—or evo-devo morphology—focuses on the morphological dimension of evo-devo [78,79] that is still important and relevant in our genocentric age because morphology represents a higher-level organization with emergent properties that cannot be reduced to genes. With the concept of the article, articulation morphology provides a conceptual framework for the empirical basis of plant morpho evo-devo. Within this framework, it investigates morphological changes in ramification and articulation in the evolution of development. The evolution of land plants involved a shift from dichotomous to lateral ramification and eventually also to axillary branching. In many cases, laterally formed articles gave rise to additional articles—for example, in compound leaves. Whereas in mainstream morphology simple and compound leaves are considered homologous organs, articulation morphology emphasizes the transformation from a single ramification in simple leaves to additional ramifications in compound leaves. What matters are the ramifications and the resulting articulation. Therefore, instead of or in addition to enquiring about the homology of organs, articulation morphology poses the more fundamental question of how ramification and articulation have changed during ontogeny and phylogeny.

Although change in ramification and articulation is central to evolution, we also have to recognize the prevalent repetition of these processes in both development and evolution. The same or similar articles, such as a particular leaf type, may be produced repeatedly along the stem and may persist over long evolutionary periods. Only some patterns of ramification and articulation change, while many others are retained over long periods.

Switching from the categorical organ-centred approach of mainstream morphology to one centred on ramification and articulation can change the questions we ask and the insights we obtain. For example, consider carpels. In mainstream morphology, a carpel is interpreted as a closed megasporophyll, that is, an organ homologous with a leaf. In contrast, according to articulation morphology, ramification at the floral apex produces a carpel primordium that develops into an article, which I prefer to call a gynoecial appendage [80]. As this gynoecial appendage develops, further ramification leads to the formation of a placenta and/or one or more ovules that produce first the nucellus and subsequently the integument(s). Thus, the formation of a carpel (a gynoecial appendage bearing ovules) involves four or five successive ramifications, resulting in four or five kinds of articles: the gynoecial appendage, the placenta, the nucellus, and one or two integuments. From this perspective, contrary to the organ-centred approach of mainstream morphology, the question in articulation morphology is no longer whether the carpel is a leaf homologue. The question is how ramification and articulation have changed. This reorientation opens a new direction for evo-devo research: instead of analyzing the carpel as a single organ, we enquire about the ramifications and the resulting articulations: where is the placenta—an article—formed? How does it ramify, and which articles result from the ramification? Because these questions concern morphology, the fundamental importance of morphology in evo-devo research becomes evident: the initial questions are framed in morphological terms, and here we have the choice between the categorical concepts of mainstream morphology, laden with problematic assumptions of homology, or the concepts of articulation morphology that refer to directly observable morphogenetic processes leading to the formation of articles.

Replacing organs with articles affects not only morpho evo-devo but also developmental genetics, which plays a central role in evo-devo besides morphology. This shift directs the analysis of gene expression and molecular networks from organs to articles, and by analyzing articles—instead of organs—new insights can be gained. For example, with regard to the gynoecium, some developmental geneticists have taken a first step in this direction. Mathews and Kramer [81] recognized “that the carpel is a complex organ consisting of a foliaceous appendage and the placenta,” that is, two articles: the foliaceous appendage and the placenta. Sattler [81] also took this first step, but from a morphological point of view, by distinguishing the gynoecial appendage from the placenta or ovule (in gynoecia with only one ovule; see also [82,83]). Articulation morphology advances this perspective further by recognizing the nucellus and the integument(s) as additional articles. This step has also been taken in the elucidation of the developmental genetics of the nucellus and integument(s) [84]. Thus, in this instance, developmental genetics and articulation morphology have begun to converge with regard to the units they investigate. However, although developmental genetics has, to some extent, begun investigating articles, the concept of the article has not been acknowledged explicitly. And to some extent, evo-devo still assumes the morphological categories of classical mainstream morphology. In contrast, articulation morphology entails a radical departure and liberation from the constraints, problems and pseudo-problems of classical, organ-centred mainstream morphology. In articulation morphology, we no longer ask whether the carpel is an organ system or an organ, or whether the placenta, the ovule and the integument(s) are an organ or part of an organ. Similarly, we no longer ask whether a stamen—especially a stamen fascicle—is an organ or organ system, or whether a compound leaf is an organ or an organ system. According to articulation morphology, all of these structures consist of articles that have differentiated in different ways. The central task, therefore, is to determine how and why they have diverged in their differentiation. Asking whether they are organ systems, organs, or parts of organs may not only be unhelpful for these investigations but may even impede progress. Traditional terms may nevertheless be retained, provided it is recognized that they do not represent categorical realities but rather serve as convenient means of communication. Kirchoff et al. [85] discussed the complexities of terminology.

Although articulation morphology is fundamentally a morphological approach, it might be integrated with molecular genetics, so that articles would be understood not only morphologically, in terms of morphogenetic processes, but also at the molecular level, in terms of gene expression and regulatory networks [26,86]. However, such integration is beyond the scope of articulation morphology as envisaged here, which is a morphological approach whose goal is to provide an empirical, dynamic, all-inclusive and unifying conceptual framework for morphology and morpho evo-devo in terms of articles rather than organs. Nevertheless, although this framework remains morphological and within the scope of morpho evo-devo, it is relevant to developmental genetics, insofar as developmental genetics relies on morphological concepts. Evidence from developmental genetics has increasingly been used for the reconstruction of evolution [86,87].

## 8. Conclusions

Like animals, plants exhibit polarity, but unlike animals, plants show open growth, which may continue throughout their lives. Open growth leads to ramification, and ramification leads to articulation: the formation of an article from each ramification and between successive ramifications. Because ramification and articulation are intrinsic to open growth, open growth constitutes the most basic and distinctive process of plant construction. It entails continuous development leading to the genesis of articles.

According to articulation morphology—which could also be called segmentation morphology—a plant is an articulated (or segmented) whole: a system of interconnected articles arising through ramification. In contrast to mainstream morphology, whose fundamental units are organ categories, articulation morphology recognizes only one fundamental unit, the *article*. An article originates as a growth centre (primordium) in continuity with its preceding article. Articles are directly observable as the result of ramifications, and, in this sense, are not controversial and provide an empirical basis. Organs, by contrast, are defined within the framework of a morphological theory and its associated homology concept, such as the classical root–stem–leaf theory of mainstream morphology, which is controversial and limited. Although articulation morphology is based on articles instead of organs, a connection to organs can be made. If an article does not ramify further, it may correspond to an organ. Thus, for example, a simple leaf is an article and also an organ. However, if a leaf produces leaflets, it corresponds to a system of articles. Articles between ramifications, such as internodes, do not correspond to an organ.

Abandoning the primacy of the traditional morphological categories may be unsettling—perhaps even painful—for morphologists accustomed to categorical thinking. However, beyond such an initial reaction lies the potential for liberation from the constraints, problems and pseudo-problems of classical mainstream morphology. Articulation morphology can accommodate all structures that deviate from the categorical, organ-centred framework of classical mainstream morphology. Thus, articulation morphology is all-inclusive: even the most deviant structures can be understood as deviant patterns of ramification and articulation.

In addition to being all-inclusive, articulation morphology is also unifying because the concept of the article applies to all plants—from algae to bryophytes and vascular plants—and across all levels of organization—from organ systems to organs to enations and hairs—whereas organ-centred mainstream morphology creates an artificial division between organs and other structures, thereby obscuring the structural unity underlying all plant construction.

Ramification can also be understood as an expression of differential growth, because differential growth may give rise to articulation. And the development of the articles can be seen as a process of differentiation. Thus, instead of referring to a plant as an articulated whole, one could conceive of it as a differentiated whole that produces articles. Instead of ‘articles,’ one could refer to segments, structural units, or simply structures defined by ramification—that is, by differential growth. Articulation can then be described as structuration: the formation of structures. However, these structures are understood dynamically as process combinations; they encompass the whole continuum from the organ system of the shoot to organs to hairs (trichomes)—hence articulation morphology is not organ-centred.

If one wishes to compare articles, one may apply fuzzy logic, according to which differences range from 0% toward 100%, which means “more or less different” in ordinary language. One may interpret the difference, which corresponds to degrees of similarity, as the homology of articles, ranging from total to partial homology, with total homology being 0% difference (sameness). However, in articulation morphology, the central and most basic concept is no longer morphological homology but transformation: the transformation of ramification and articulation. This changes the most basic questions we ask. Instead of asking questions about morphological homology, we ask how ramification and articulation have changed during development and evolution—a shift in focus that fundamentally changes our way of thinking about morphology and, consequently, morphological investigation in morpho evo-devo. Thus, articulation morphology investigates transformation directly, without the interference of morphological homology, whereas mainstream morphology uses morphological homology as its most basic concept and infers the transformation of development during evolution within that framework, with all its unresolved problems and controversies.

Throughout the history of plant morphology, a wide range of morphological theories and concepts has been proposed [12], and their proponents have often been more or less critical, or even hostile, toward each other. More recently, however, at least some morphologists have become more tolerant, viewing different approaches as complementary rather than mutually exclusive [4,5]. Articulation morphology also recognizes this complementarity, even with classical mainstream morphology. However, it goes further by offering a factual, non-controversial foundation that can serve as a common ground for all morphologists and evo-devo biologists, regardless of their theoretical preferences.

Briefly, the significance of articulation morphology is at least two-fold. First, it transcends the rigidity of the categories of classical mainstream morphology that may create pseudo-problems. Second, it introduces an empirical, dynamic, all-inclusive, and unifying approach: empirical and dynamic because it is based on the observable processes of open growth, ramification and articulation; all-inclusive because it includes all exceptional structures (misfits), which thereby cease to be exceptions; and unifying because it implies the concept of the article—a structural unit that applies to all plants, from algae to bryophytes and vascular plants, and across all levels of morphological organization—but this concept has been largely overlooked because of the organ-centered approach of mainstream morphology. Articulation morphology thus highlights the fundamental significance of the article for plant morphology and plant evo-devo. It provides a conceptual framework for plant morpho evo-devo that is also relevant to the developmental genetics of plants.

Articulation morphology can also be called *open morphology* because it is based on the open growth of plants. However, regardless of the name chosen, the proposed approach embodies some of the most fundamental ideals of science: science strives to be empirical, which means evidence-based; because nature is fundamentally dynamic, science also seeks to be dynamic; and it aims to be all-inclusive and unifying. These principles play a central role in physics, widely regarded as the most advanced science, and they are also embodied in articulation morphology.

For updates to this paper, see: Sattler, R. Articulation Morphology of Plants and Plant Evo-Devo. https://beyondwilber.ca/about/EvoDevo/Articulation-Morphology.html (accessed on 25 February 2026).

## Data Availability

Not applicable.
